# Domino-liver transplantation: toward a safer and simpler technique in both donor and recipient

**DOI:** 10.1007/s13304-020-00886-4

**Published:** 2020-09-23

**Authors:** Jan Lerut, Maxime Foguenne, Quirino Lai, Jean de Ville de Goyet

**Affiliations:** 1 Institute for Experimental and Clinical Research (IREC), Université Catholique de Louvain (UCL), Avenue Hippocrate 55, 1200 Brussels, Belgium; 2 Liver Transplant Program, Sapienza University of Rome, Rome, Italy; 3 University Pittsburgh Medical Center (UPMC-Italy) at the ISMETT (Istituto Mediterraneo for Trapianto e Terapie ad Alta Specializzazione), IRCCS, Palermo, Italy

**Keywords:** Liver transplantation, Domino-liver transplantation, Venous allograft, Familial amyloidotic neuropathy

## Abstract

Domino-liver transplantation represents a rare chance to expand the donor liver pool. Fear of putting both donor and recipient at disadvantage has meant that the procedure has not been applied universally. A modification of the original technique which allows both safe procurement of the graft as well as safe implantation of the reconstructed graft in the domino-graft recipient using a 180° rotated, adequately trimmed, free iliaco-caval venous graft is described in detail.

## Introduction

Domino (or sequential) liver transplantation (DLT) represents an opportunity to expand the liver allograft pool. The first Swedish experiences with liver transplantation (LT) for hereditary transthyretin amyloidosis, also known as familial amyloidotic polyneuropathy (FAP), the high incidence of this disease in Portugal and the structural normality (except for the production of the mutant TTR) of such livers, led to the development of DLT [1–3]. The concept is based on the knowledge that non-cirrhogenic, liver-based, metabolic diseases such as FAP, maple syrup disease, hyper-homocysteinemia, methylmalonic acidemia, and hypercholesterolemia are not or slowly transmitted to recipients which do not have these inherited traits [4, 5]. Up to December 2019, 2217 LTs were reported to the FAP World Transplant Registry, the Domino-liver Transplant Registry included 1210 (54%) DLT. DLT is normally only used in elderly and/or cancer patients [6, 7]. The non-use of these potentially excellent liver allografts has four reasons: (a) ethical concerns about putting the domino-donor at risk of a more complex surgical procedure and the domino-recipient for a possible disease transmission (reported in 3.3–21% of domino-recipients); (b) fear of technical complications related to a more difficult arterial inflow and venous outflow reconstruction of the domino-allograft; (c) increased logistics linked to the organization of two simultaneous, LT procedures, and finally (d) the (intra-operative) discovery of advanced liver fibrosis (‘cardiac liver’) caused by the underlying amyloidotic cardiopathy [8–14].

Over the years, technical developments, routine introduction of inferior vena cava (IVC) sparing hepatectomy techniques, and accumulated experiences in centers specializing in liver-based metabolic diseases overruled these concerns [15–20]. The original ‘Coimbra-procedure’, described by A. Furtado in 1992, including total hepatectomy with the removal of the IVC, extensive dissection of the supra-hepatic IVC at or *above* the diaphragmatic ring to assure a sufficiently long supra-hepatic IVC cuff, and the systematic use of the veno-venous bypass (VVB) to overcome hemodynamic instability caused by IVC clamping in neurologically dysregulated FAP patients, clearly disadvantaged the domino-donor compared to the ‘classical’ liver transplant patient [2, 3]. Ten years later, the Lisbon Rui Cabral team, led by JR. Pena and E. Barroso, introduced the ‘double piggy-back’ implantation technique in which the IVC was preserved and the use of VVB avoided in both domino-donor and domino-recipient [16, 21–23]. The growing confidence in DLT has been exemplified by its extension to split- and living donor DLT [24–27]. A further modification of the technique, aiming at making DLT safer and simpler, is presented here in detail.

## Surgical technique (Fig. 1a–d)

DLT is performed only following informed consent obtained from both domino-donor and domino-recipient patients after a detailed explanation of both donor and recipient transplantation procedures. DLT was approved by the ethical review board of the Université Catholique de Louvain.

**Fig. 1:**
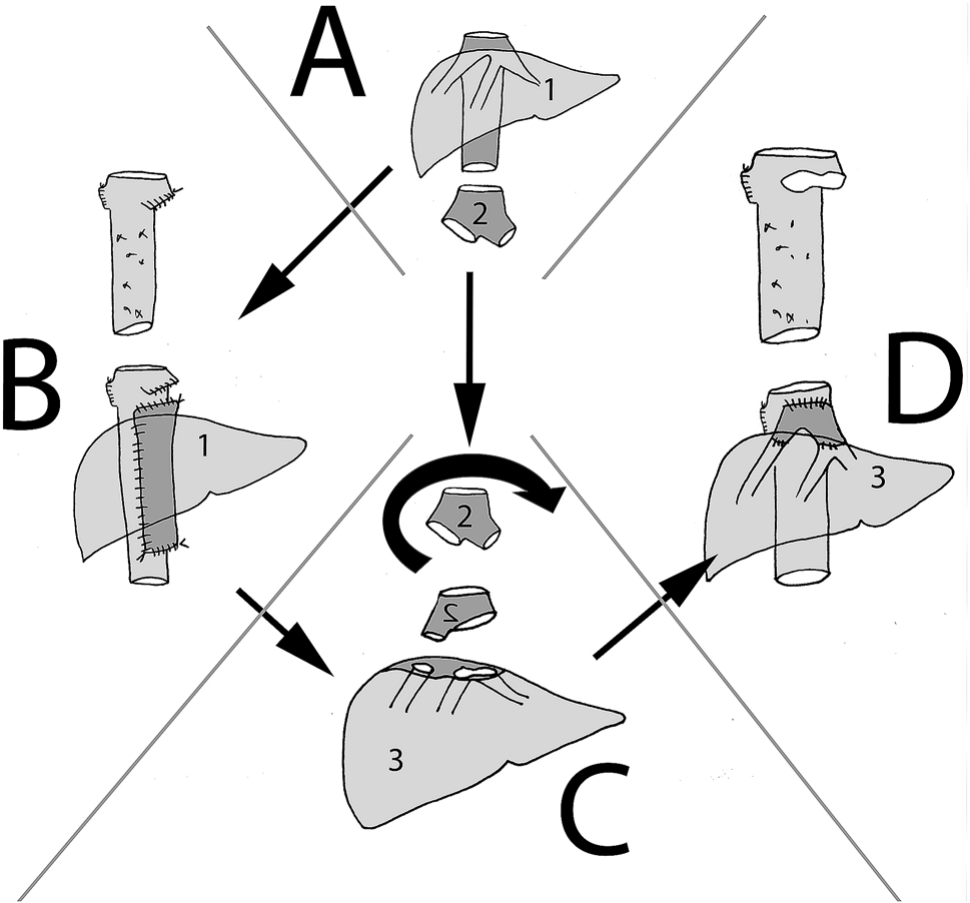
Domino-liver transplantation technique. **a** The liver (1) is retrieved from a post-mortem donor together with the iliaco-caval bifurcation (2) of the same donor as a free vascular graft. **b** In the first recipient or domino-donor, the native vena cava is preserved; the native (or domino) liver (3) is explanted the hepatic veins being cut flush to the liver parenchyma. Depending on their anatomical variation, left and median hepatic veins are either together or separated. The post-mortem allograft (1) is implanted using a large latero-lateral cavo-cavoplasty. **c** On the back table, the iliaco-caval homograft is rotated over 180° (2).The iliac part of this graft is sutured to the right hepatic vein; the caval part to the joined middle and left hepatic veins of the domino-liver (3). **d** In the second recipient or domino-recipient, the vena cava has been preserved and the domino-liver (3), extended by the tailored venous homograft (2), is anastomosed in a piggy-back manner onto the cuff of left and middle hepatic veins

### The domino-donor operation

The liver is rolled off from the IVC and prepared from below upwards after ligation of all hepatic veins draining segment I and of any larger right inferior (Makuuchi) veins. The right hepatic vein (RHV) and the cuff of middle (MHV) and left hepatic (LHV) veins are isolated separately. Suture ligation and transection of the left phrenic vein allow lengthening of the MHV–LHV cuff. Grasping the tendinous ring of the diaphragm with a Babcock clamp makes the dissection of the supra-hepatic IVC easier, because it better exposes the dissection plane. The diaphragmatic portion of the supra-hepatic IVC and the para- and retrocaval areas are left intact. The dissection of the hilar structures is guided by the pre-transplant angio-CT scan, which is done in all domino-donors and domino-recipients to get accurate information about existing vascular variations. After dissection of the proper, common, and gastroduodenal arteries, these arteries are bull-dog clamped and the proper hepatic artery is divided at the bifurcation of gastroduodenal and common hepatic arteries in such a way that a small Carrel patch can be obtained. Small (left) accessory arteries are ligated. In case of separate right and left hepatic arteries, these will need to be implanted separately. The portal venous trunk is divided in its middle and the bile duct is transected just above the level of the cystic duct. The RHV is transected vertically using an endovascular stapler (United States Surgical Corporation, Norwalk, Connecticut, USA). Precise stapler application close to the liver parenchyma permits a rapid, safe, and tight transection of the RHV without narrowing the IVC.

Moreover, this maneuver avoids bleeding from the parenchymal side when extensively mobilizing the liver and allows the liver to be rolled off further from the IVC, helping in the safe isolation of the MHV and LHV. Broad experience with this type of vascular closure in classical LT and living donor LT revealed that the removal of a precisely placed stapler line allows obtaining a clean, rectilinear, and longer RHV stump. Indeed, in case the RHV is closed using a running suture, the varying and thicker amounts of a vascular wall comprised in such suture frequently oblige to resect a larger vessel rim. Consequently, the RHV may be shorter.

The inferior RHV, present once in these series, was simply suture-ligated even if larger than 5 mm.

The isolated M-LHV cuff is double clamped with a large right-angled vascular clamp. After scissor transection, the distal clamp is removed, and the cuff is closed using running sutures. The post-mortem allograft is implanted in the domino-donor using a large latero-lateral cavo-caval anastomosis under partial IVC clamping using the Lerut–Satinsky vascular clamp (Ulrich-AG, St.Gallen, Ch). Its prolonged vertical and horizontal part allows a safe lateral clamping of the IVC and so a comfortable suturing of donor and recipient IVCs. All other vascular and biliary sutures are done as usual [17].

### The domino-back-table operation (Figs. 1, 2a–d)

The exsanguinated liver is flushed with HTK or IGL solution through the cannulated short portal vein. The RHV stapler line is taken down and the liver gently massaged to allow rapid emptying of its blood content. Depending on the anatomical variation, LHV and MHV are together or separated. Venoplasty (if close to each other) or use of a small venous patch (if too far from each other) allow one single orifice to be created [28]. The easiest way to reconstruct correctly the supra-hepatic outflow tract of the graft is to anastomose the orifices of RHV and of the M-LHV cuff of the domino-graft to a modified free iliaco-caval vein graft (originating, if possible, from the same post-mortem donor). The anatomical display of the common iliac vein bifurcation is of importance: the right vein is shorter, somewhat larger and runs more vertically; the left one is longer, somewhat smaller, and deviates at a sharper angle (Fig. 1). After rotating this venous graft by 180°, the graft is trimmed to length. The IVC is cut in a slightly oblique fashion and its length reduced to 1.5–2 cm. The left iliac vein is cut one to 1.5 cm from i confluence with the IVC; by doing so, the diameters of the left iliac vein and the RHV from the domino-graft will match exactly. When cutting the right common iliac vein flush to its merger with the IVC in an upwards and slightly oblique direction, its diameter exactly matches the one of the common M-LHV cuff.

**Fig. 2:**
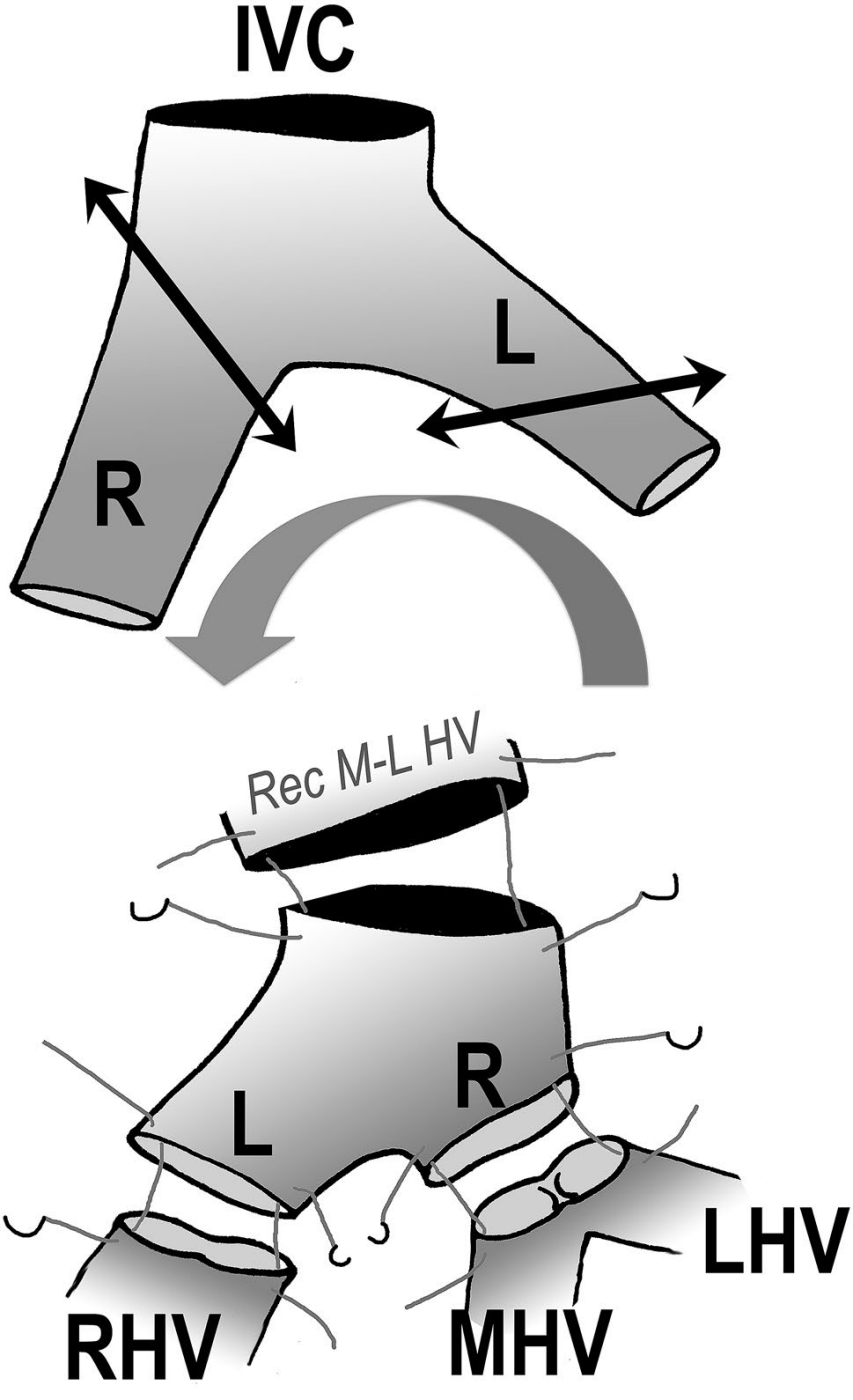
Adaptation of the 180° rotated iliaco-caval venous graft to the hepatic venous anatomy of the domino-graft

### The domino-graft recipient operation (Figs. 3d, 2d)

The diameters of the newly constructed supra-hepatic cuff and the M-LHV cuff of the domino-recipient will fit, thereby allowing a safe piggy-back implantation of the domino-graft. The length of the IVC interposition graft needs to be trimmed ‘in situ’ in such a way that kinking, and outflow obstruction are avoided. All anastomoses are performed using running polypropylene 5 to 7/0 sutures. This type of implantation can be done in the domino-graft recipient without total IVC clamping and so without VVB use.

**Fig. 3:**
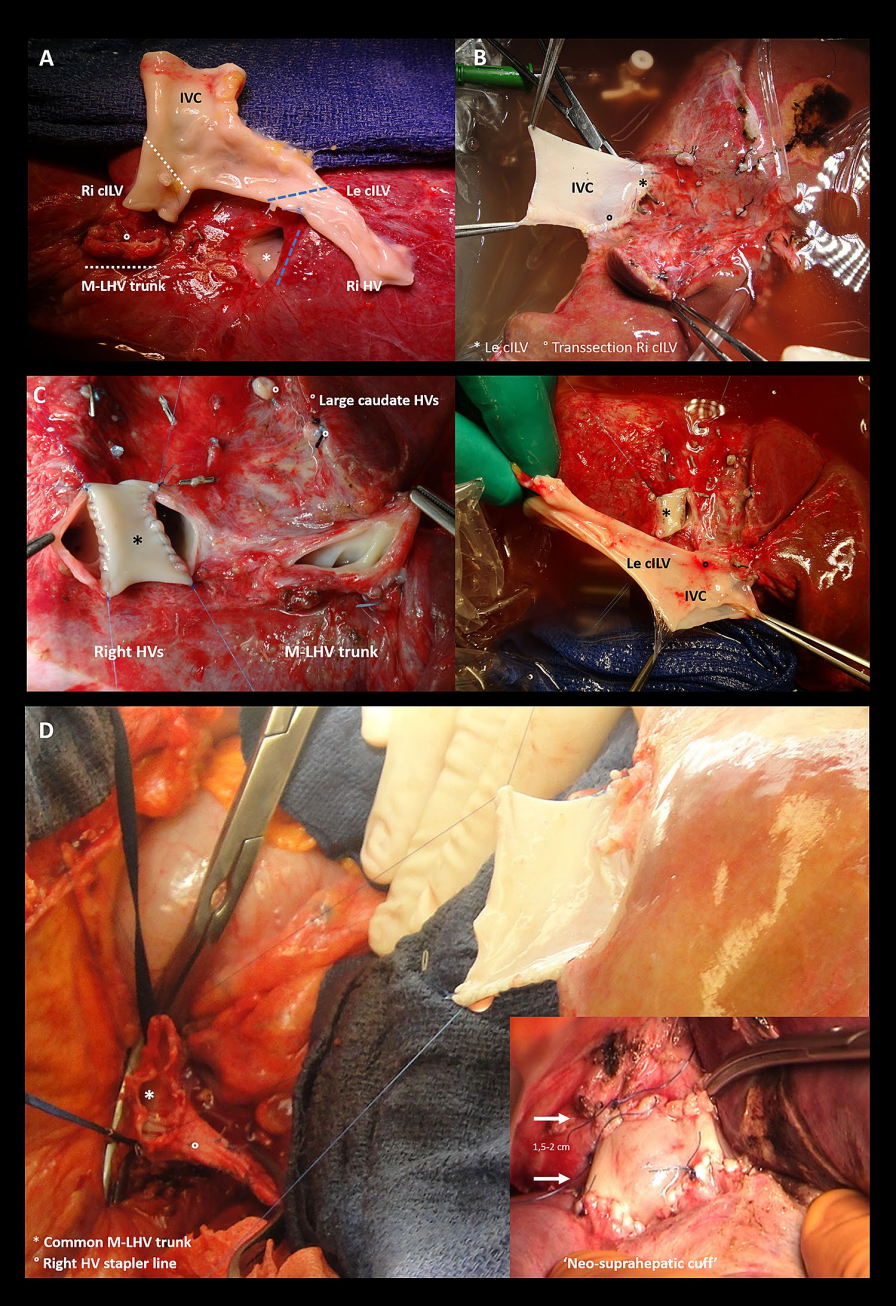
**a**–**d** Intraoperative view of back-table outflow reconstruction of the domino-graft. **a** Procured domino-liver with the hepatic veins cut flush to the liver parenchyma and the ready to use iliaco-caval venous graft. **b** Back-side of the reconstructed domino-graft. The obliquely cut, IVC orifice (arrow) exactly matches the diameter of middle and left hepatic vein (M-LHV) cuff as does the diameter of the left iliac and the right hepatic (RHV) veins. **c** Sometimes a patch (asterisk) is needed to join distant hepatic vein orifices. **d** Adequately shortened neo-supra-hepatic caval cuff

## Results

During the period February 2000–January 2017, 16 LT were performed for FAP. On three (18.7%) occasions, DLT was not possible due to extended intrahepatic and extrahepatic portal vein thrombosis (1 pat) and advanced fibrosis (2 pats) due to autoimmune cirrhosis and restrictive amyloidotic cardiomyopathy. One Fap liver was split for two small adults. Indications for DLT were hepatocellular cancer (7 pats), primary biliary cholangitis (3 pats), alcoholic cirrhosis with severe encephalopathy (3 pats), and neuro-endocrine metastases (1 pat). IVC-sparing hepatectomy without VVB use was performed in all LT procedures. Median warm ischemia time of the domino-graft was 43 min (range from 30 to 73). In one case, the hepatic artery had to be anastomosed to the splenic artery because of fibrotic changes of the hepatic artery caused by trans-arterial chemo-embolisations; in one right and left, hepatic arteries were anastomosed to similar, corresponding recipient arteries, and in another, the portal vein needed to be prolonged using a free iliac vein to overcome an extended portal vein thrombosis. The day-7 angio-CT scan of the patient in which a dominant inferior RHV was not implanted revealed a normal venous outflow of the right liver. In all domino-grafts, Doppler ultrasound revealed normal, triphasic, outflow patterns, and angio-CT showed a well-vascularized segment I. Two domino-donors presented a right pleural effusion. None of the domino-graft recipients presented after a median follow-up of 40 months (range 1–206) signs of disease transmission. One domino-liver recipient developed an ascitic decompensation 10 years post-DLT due to fibrotic changes of the neo-supra-hepatic cava cuff; a single balloon dilatation resolved this problem. In this case, a stored iliaco-caval graft from another donor had been used to reconstruct the venous outflow. Four domino-recipients died of recurrent cancer (at 7, 23, 41,159 mo post-DLT), two of cardiac complications (at one and six mo), one each of pulmonary fibrosis (at 152 mo), suicide (39 mo), and massive variceal bleeding (1 mo). There was no documented disease transmission in any of the domino-graft recipients.

## Discussion

**Fig. 4:**
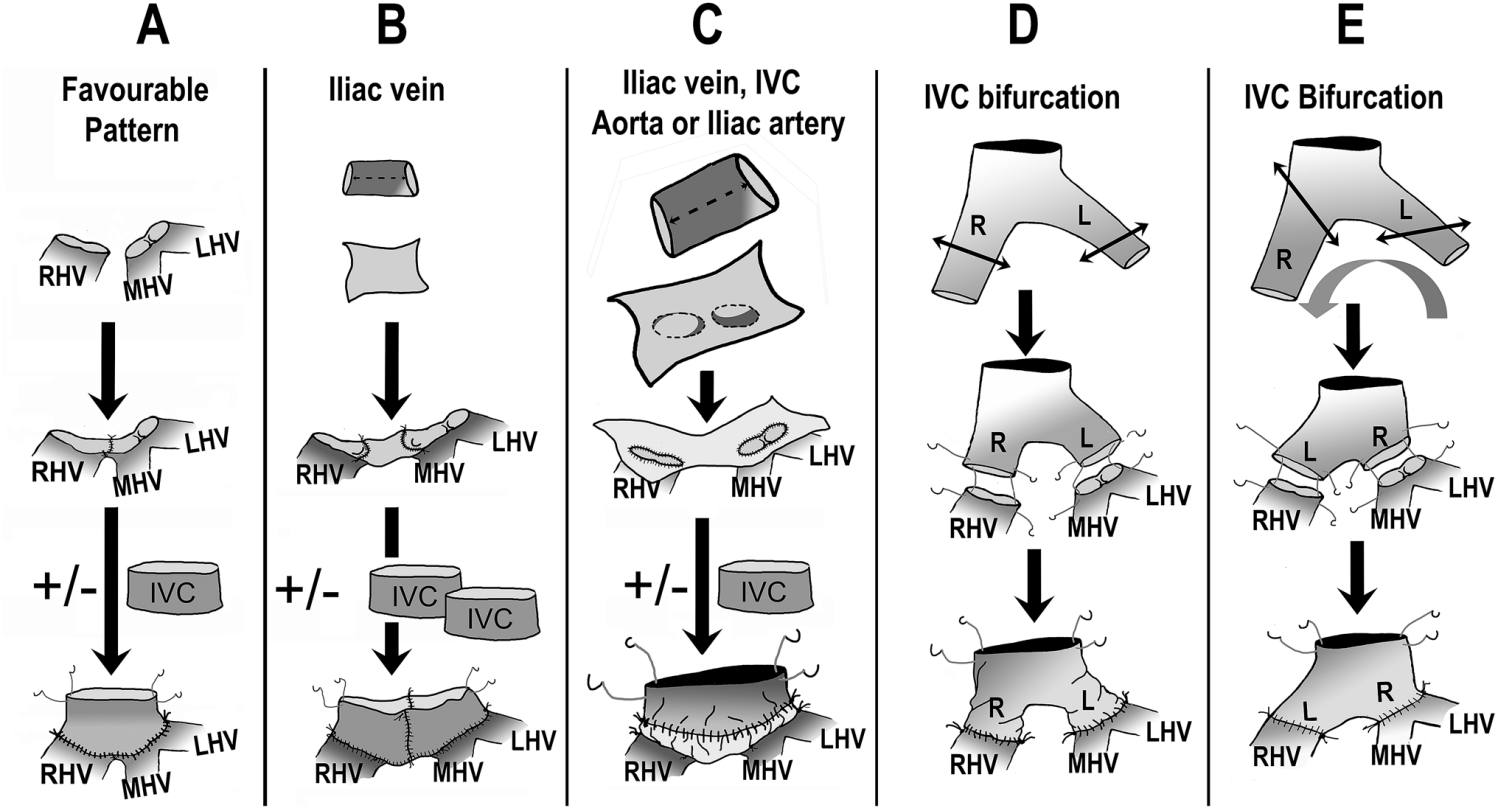
**a**–**e** Schematic representation of different, published, types of “neo-caval” outflow reconstruction of the domino-graft. The here presented technique (**e**) compares favorably with the others, because avoiding possible problems caused by a too short (**a**–**b**) and or too long (**d**) outflow tract or because being simpler than techniques (**a**–**d**)

Domino LT represents a rare opportunity to enlarge the liver donor pool [2]. The extensive supra-hepatic IVC dissection aimed at obtaining a long supra-hepatic venous cuff (but possibly causing pleural and pericardial effusions) and the systematic use of VVB aiming at overcoming hemodynamic instability when clamping the IVC (but possibly causing cutaneous (wound infection and lymphocele), venous (pulmonary and peripheral embolism), and neurological (neuropraxia, paresthesia) complications disadvantaged the domino-donor compared to the ‘classical’ liver recipient. The domino-recipient was also disadvantaged by a more challenging graft implantation due to insufficient vessel lengths. Technical improvements in both domino-donor and domino-recipient procedures allowed the ethical concerns concerning the applicability of DLT to be overcome [6]. Routine use of IVC-sparing hepatectomy techniques and attention to the domino-graft outflow reconstruction played a major role in this [15, 18]. Many variants have been reported to optimize the domino-graft venous outflow (Table 1; Fig. 4). The extension of the supra-hepatic vena cava cuff by a free venous graft, reported by Azoulay et al. in 1999, was a first step to make the DLT easier [29]. In 2001, Nishida et al. proposed the end-to-side infra-hepatic cavo-cavostomy as a means to overcome the difficulty of the supra-hepatic anastomosis; the supra-hepatic IVC was closed with a vein patch [30]. At the 2001 ESOT congress, the Lisbon team reported for the first time the use of FAP-livers with IVC preservation in the FAP-donor and extension of the domino-graft with a free iliaco-caval graft [21, 22]. Pinto-Marques et al. confirmed, in a series of 260 DLT, the feasibility without VVB use and with outflow tract reconstruction (“neo-supra-hepatic cuff”) of the domino-allograft using IVC, iliaco-caval, reno-caval, or pulmonary veins. It is of note that eight patients presented a venous outflow obstruction and that six patients needed to be re-transplanted [16]. The fact that 16 more technical modifications have been reported during the period 2001–2019 indicates the need for further standardization and simplification of the technique [8, 28, 31–45]. The venous outflow reconstruction of the domino-graft, such as described here, is in line with this. The use of a 180° rotated and anatomically-based modified free iliaco-caval vein graft allows the procedure to be simplified. When adequately tailored and incised, this venous graft does not need any supplementary gesture. In contrast to the literature, none of our domino-recipients developed a post-transplant venous outflow obstruction related to the design of the neo-supra-hepatic venous cuff. Interference with renal and thoracic organ procurement teams is eliminated as there is no need to take the pulmonary vein nor reno-caval venous confluence and the piggy-back implantation of the domino-graft is easy. The implantation of a post-mortem whole or right split liver graft in the domino-donor is also easy when performing a side-to-side cavo-caval implantation under partial clamping of the recipient IVC. Our extensive experience with this technique also learned that implantation of right accessory, even dominant, HVs looks not to be necessary for full-size LT [17].

**Table 1:** Different reconstruction outflow techniques reported in case of domino liver transplantation

Author	References	Year	Country	*N*	Type outflow reconstruction domino graft	Remark/ Reported Complications
Azoulay	[29]	1999	France	10	IVC preserved (5 patients) with straightforward cavo-caval anastomosis IVC not preserved (5 patients) because of technical difficulties; upper cavo-caval anastomosis difficult in 2 patients due to short caval stump.	Temporary renal insufficiency (combined liver-kidney transplantation): 1 FU: 2–22 months
Nishida	[30]	2001	USA	5	Donor venous patch (2 patients) and infra-hepatic end-to-side IVC anastomosis	No complications FU: 5–50 months
Pacheco-Moreira	[31]	2003	Brazil	1	Donor IVC with common ILVs. Separate anastomosis to RHV and M-LHV trunk	FU: 2 weeks
Jabbour	[32]	2006	USA	1	Donor IVC with (2-cm segment) common ILVs Common ILVs separately anastomosed to RHV and M-LHV common trunk (5/0 polypropylene)	FU: none
Garcia	[33]	2006	Brazil	1	Donor IVC with common ILVs used as Y-shaped vascular graft. Common ILVs anastomosed to RHV and M-LHV trunk (5/0 polypropylene)	VVB not used No complication FU: 8 days
Cerqueira	[34]	2006	Brazil	1	Recipient Portal Vein inverted bifurcation as interposition graft: R and LPV to RHV and to common M-LHV trunk (5/0 polypropylene)	No complication FU: 2 months
Cescon	[35]	2007	Italy	3	Donor IVC with one common ILV opened longitudinally. Quilt plasty including all HVs and Caudate lobe HV. The inferior wall opened circularly to be anastomosed to each venous orifice. The external edge trimmed to obtain a cylinder which is anastomosed end-to-end to the recipient cuff formed by all 3 HVs. All sutures are everting with 5/0 and 6/0 polypropylene	Segment I vein included in reconstruction FU: 2 weeks No complications
Mergental	[36]	2007	Netherlands	1	Recipient obliterated Umbilical Vein as interposition graft	NA FU: 3 months; anticoagulation
Lacerda	[37]	2008	Brazil	3	Donor IVC with (3-cm segment) common ILVs RILVs anastomosed to RHV and LILV to M-LHV trunk (6/0 polypropylene). The IVC anastomosed to all 3 recipient HVs	No complications Deadly cardiac arrest day 1 FU: NA
Liu	[38]	2008	Taiwan	1	Donor IVC with common ILVs longitudinally opened to become a venous patch. A midline incision of the vein patch was made to allow anastomosis with all 3 HVs joined previously (6/0 polypropylene). The external edges of the vein patch were further fashioned to be a wide cuff	Domino graft with long HV stumps; recipient IVC totally clamped; no VVB use Biliary leakage FU: 6 weeks
Escobar	[23]	2009	Spain	36	Donor infra-and retro-hepatic IVC to perform patch with 3 HVs Both in case of IVC preserving or resecting hepatetcomy	VVB use in 20 patients Incidence of minor cardiovascular events, acute renal dysfunction and outcome similar in IVC preservation and IVC resection with VVB use in FAP patients FU: up to 26 months
Suarez-Munoz	[39]	2009	Spain	1	Separate Donor iliac veins longitudinally opened and superimposed	All 3 HVs domino-graft joined No complication FU: 10 days
Soin	[40]	2010	India	1	Cryopreserved PV Bridge venoplasty between RHV and M-LHV trunk	Living donor LT procedure Total occlusion IVC domino-recipient FU: 3 months
Padín	[41]	2011	Argentina	1	Donor iliac vein longitudinally opened, leaving a vascular rim of ~ 1.5 cm. The internal edge of the rim anastomosed to the common orifice of the joined HVs (6/0 polyproylene). The lateral edges of the venous iliac graft sewn to turn the rim into a cylindrical structure obtaining a ‘neo-supra-hepatic’ IVC	All 3 HVs domino-graft joined NA FU: none
Llado	[42]	2014	Spain	1	Donor arterial graft using both iliac arteries	No complications
Pinheiro	[28]	2014	Belgium	1	Donor ILV graft longitudinally opened. 2-cm ‘diamond patch’ as a bridge between M-LHV trunk and RHV (6/0 polypropylene sutures). Running suture of the iliac vein graft encompassing all HVs and the patch followed by suturing the lateral walls of the iliac vein fence to each other to obtain a IVC cylinder	No complications FU: 6 years
De la Serna	[8]	2015	Spain	39	Cavo-caval side-to-side anastomosis (6 patients); end-to-side anastomosis without graft (16); end-to-side anastomosis with venous interposition (IVC: 4, auricle: 2; Y-shaped IVC-iliac bifurcation: 5) and arterial graft interposition (aorta: 1, “Bellvitge” arterial graft: 5) grafts	Subacute Budd-Chiari Syndrome at median delay of 7 months: 4 (all venous graft) treated by balloon dilatation and stenting FU: 81 ± 53 months
Pinto Marques	[16]	2015	Portugal	114	Donor IVC-left renal (50 patients); IVC (41); IVC-iliac (20) and pulmonary (3) donor vein grafts to create a “neo-supra-hepatic” cuff	Outflow obstruction after piggy-back: 8; re-LT: 6
Cepeda-Franco	[43]	2017	Spain	1	Donor single ILV graft cut in length to fit distance of all 3 HVs and longitudinally split at the lower and upper side to fit the HVs diameter and M-LHV trunk	No complication FU: 18 months
Herden	[44]	2019	Germany	2	Donor iliac vein for plastic reconstruction of the separate openings of the 3 HVs followed by side-to side triangular caval anastomosis	No complications FU: some months
Lerut	–	2020	Belgium	12	Donor iliaco-caval confluence anatomically tailored to HV orifices	RHV obstruction 10 years after uneventful post-LT course FU: 6–206 months

*Ref* reference, *N* number, *IVC* inferior vena cava, *FU* follow-up, *ILV* Iliac veins, *R/M/LHV* right/middle/left hepatic vein, *VVB* veno-venous bypass, *NA* not available, *LT* liver transplantation

## Conclusion

The technical simplification of DLT described here addresses the challenge of the outflow reconstruction, the avoidance of VVB use in both domino-donor and domino-recipient, and possible interference with thoracic and renal procurement procedures related to venous graft harvesting. The modified outflow reconstruction of the domino-graft should represent a standardized approach to use in domino-liver transplantation, aiming thereby at optimal use of these precious grafts.

## Abbreviations

DLT: Domino-liver transplantation
FAP: Familial amyloidotic polyneuropathy
LT: Liver transplantation
MHV, LHV, RHV: Middle, left, right hepatic vein
IVC: Inferior vena cava
VVB: Veno-venous bypass
